# Investigation of the Ferredoxin’s Influence on the Anaerobic and Aerobic, Enzymatic H_2_ Production

**DOI:** 10.3389/fbioe.2021.641305

**Published:** 2021-02-26

**Authors:** Jamin Koo, Yeeun Cha

**Affiliations:** Department of Chemical Engineering, Hongik University, Seoul, South Korea

**Keywords:** ferredoxin, biohydrogen, metalloprotein, redox biochemistry, oxidation

## Abstract

Ferredoxins are metalloproteins that deliver electrons to several redox partners, including [FeFe] hydrogenases that are potentially a component of biological H_2_ production technologies. Reduced ferredoxins can also lose electrons to molecular oxygen, which may lower the availability of electrons for cellular or synthetic reactions. Ferredoxins thus play a key role in diverse kinds of redox biochemistry, especially the enzymatic H_2_ production catalyzed by [FeFe] hydrogenases. We investigated how the yield of anaerobic and aerobic H_2_ production vary among the four different types of ferredoxins that are used to deliver electrons extracted from NADPH within the synthetic, fermentative pathway. We also assessed the electron loss due to O_2_ reduction by reduced ferredoxins within the pathway, for which the difference was as high as five-fold. Our findings provide valuable insights for further improving biological H_2_ production technologies and can also facilitate elucidation of mechanisms governing interactions between Fe–S cluster(s) and molecular oxygen.

## Introduction

Ferredoxins (Fd) are redox proteins that mediate electron metabolism in numerous kinds of cells across diverse organisms. The [Fe–S] cluster(s) in ferredoxins are responsible for electron receipt and transfer to redox partners. The type and number of [Fe–S] clusters in a ferredoxin can vary, which in turn affect the rate at which electrons are delivered, as well as redox potential of the protein (Guan et al., 2018). For example, *Synechocystis* sp. PCC 6803 ferredoxin (SynFd) has one [2Fe–2S] cluster while *Clostridium pasteurianum* ferredoxin (CpFd) harbors two [4Fe–4S] clusters. The midpoint redox potentials for the two are −412 and −387 mV, respectively (Bottin and Lagoutte, 1992; Brereton et al., 1999).

One of ferredoxins’ redox partners is a metalloprotein called hydrogenase. Hydrogenases can receive electrons from reduced ferredoxins (Fd^*red*^) and combine them with protons to produce H_2_. The enzymatic activity of hydrogenases, especially the [FeFe] subtype proficient in H_2_ production such as the one from the *C. pasteurianum* (CpI), can be exploited for biological H_2_ production (Lu and Koo, 2019); wide deployment of the technology can contribute to reduction of CO_2_ emission while supplying H_2_. Toward this aim, researchers have successfully developed both fermentative and photosynthetic pathways for reducing ferredoxins, which can subsequently be used for the enzymatic H_2_ production (Smith et al., 2011; Yacoby et al., 2011). To date, however, people have tested H_2_ production from these pathways only in an anaerobic reactor due to the high O_2_ sensitivity of [FeFe] hydrogenases.

O_2_ is a byproduct of photosynthesis and also an effective reagent for regenerating adenosine triphosphate (ATP) during fermentation. As such, it is an unavoidable constituent of the aforementioned biological H_2_ production processes; its influence on electron flow and H_2_ production need to be understood. Previous work showed that Fd^*red*^ is capable of reducing O_2_ into superoxide and peroxide (Allen, 1975), which is known as the Mehler reaction within the photosynthetic pathways. Hosein and Palmer also reported the Fd oxidation by O_2_ and its autocatalytic nature (Hosein and Palmer, 1983). Since Fd^*red*^ is the source of electrons for the enzymatic H_2_ production, presence of O_2_ results in a lower H_2_ yield via decreasing the amount of electrons delivered to the hydrogenases (Benemann et al., 1973; Koo and Swartz, 2018). O_2_ also drives inactivation of the hydrogenases, which is irreversible and fast (within minutes under the atmospheric [O_2_]) for most [FeFe] kinds (Lu and Koo, 2019). By fusing ferredoxin to an [FeFe] hydrogenase, Eilenberg et al. (2016) successfully increased the photosynthetic H_2_ production in the presence of O_2_, proposing that Fd^*red*^ delayed inactivation of the hydrogenase at the expense of electrons. The authors also confirmed the increase in aerobic H_2_ production by fusing different types of ferredoxin and hydrogenase (Koo, 2020).

We developed physiological assays and analytical methods for characterizing O_2_ sensitivity of [FeFe] hydrogenases during H_2_ production (Koo et al., 2016). Using this method, the authors studied the enzymatic H_2_ production in the presence of O_2_ at a greater detail to better understand its implications for biological H_2_ production and photosynthesis. First, we used isotopically labeled O_2_, (^18^O_2_) to confirm O_2_ reduction by Fd^*red*^. We then analyzed how the rates of O_2_ reduction vary with varying concentrations of reactants, as well as with different types of ferredoxins harboring different kind and number of Fe–S clusters. Lastly, we studied how electron loss from Fd^*red*^ to O_2_ varies among different combinations of ferredoxin NADP^+^ reductases (FNR) and ferredoxins with the goal of identifying the most effective combination in minimizing the electron leakage during the enzymatic H_2_ production.

## Materials and Methods

### Protein Expression and Purification

SynFd, CpFd, *Zea mays* Fd (ZmFd), *Anabaena variablis* Fd (AnFd), *Synechocystis* sp. PCC 6803 FNR (SynFNR), and Rice root FNR (RrFNR) were expressed *in vivo* in *Escherichia coli* and purified as described previously (Lu et al., 2015). CpI was also expressed heterologously in *E. coli* and purified as reported in the previous work (Koo and Swartz, 2018).

### Reagent Preparation

Nicotinamide adenine dinucleotide phosphate (NADPH), dithionite, glucose-6-phosphate (G6P), G6P dehydrogenase (G6PD), superoxide dismutase (SOD) and catalase were purchased from Sigma-Aldrich.

### O_2_ Reduction and Aerobic H_2_ Production Measurements

The O_2_ reduction experiments were conducted in 8.4 mL crimp vials where 840 μL reaction mixtures contained the following (unless stated otherwise): 50 mM Tris buffer pH 7.0, 10 mM G6P, 4 units of G6PD, 5.0 mM NADPH, 5.0 or 50 μM FNR, and 5.0 μM of Fd. The reaction mixtures were prepared inside a N_2_-only glovebox (CHOA Engineering). Before sealing the vials with rubber septa, magnetic stir bars were added for mixing. After removal from the glovebox, the sealed vials were placed on a stir plate to initiate mixing at 300 rpm. O_2_ was introduced to the headspace at *t* = 0 min (or 10 min when examining various pairs of FNR and Fd) by using a syringe with a 20-gage needle (Daehan Sciences); air or a gas-tight handheld tank filled with O_2_ was used as a source. O_2_ and H_2_ concentrations were measured by sampling 200 μL of the headspace with a valved 23-gage needle (Daehan Sciences), and using gas chromatography (GC 6500, YL Instrument). The H_2_ production experiments were done in the same manner with the addition of 10 nM CpI to the aforementioned reaction mixtures inside the anaerobic glovebox.

### ^18^O_2_ Reduction Experiments

The amount of H_2_^18^O formed by the reaction mixtures (50 mM Tris buffer pH 7.0, 10 mM G6P, 4 units of G6PD, 5.0 mM NADPH, 5.0 or 50 μM FNR, 5.0 μM of Fd, 4.2 U SOD and 4.2 U catalase) were measured as follows. The 8.4 mL crimp vials (Thermofisher Scientific) were first opened using a decapper, and the reaction buffer were immediately put in the 80 °C water bath (Daehan Sciences) for the following hour to terminate any ongoing enzymatic reactions. Next, the reaction buffer was distilled at 120 °C and only the water vapor was collected by condensation. The collected samples were mixed with distilled H_2_O at two different ratios (2- and 10-fold dilution) and submitted together with a sample containing only distilled H_2_O to the mass spectrometry (National Center for Inter-University Research Facilities, Seoul) for analysis of H_2_^18^O content.

## Results and Discussion

### Confirmation of ^18^O_2_ Reduction by Fd^*red*^

**FIGURE 1:**
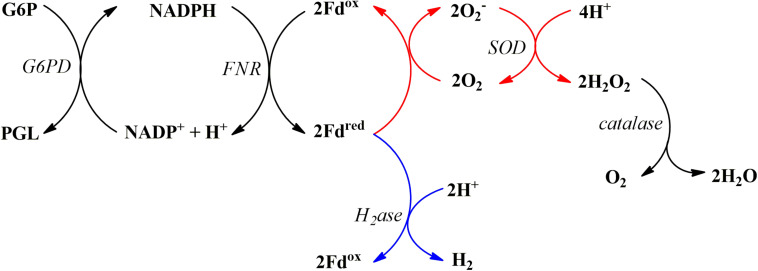
The biochemical reaction series for NADPH-driven reduction of Fd, putative subsequent reduction of O_2_ into O_2_ radical, and the final conversion into H_2_O and O_2_ via SOD and catalase (red arrows); the electron flow for subsequent H_2_ production are shown in blue arrows. Abbreviations are as follows: glucose-6-phosphate (G6P), glucose-6-phosphate dehydrogenase (G6PD), 6-phosphoglucono-D-lactone (PGL), hydrogenase (H_2_ase).

We have previously reported O_2_ consumption by SynFd^*red*^ during fermentative H_2_ production where electrons are extracted from NADPH and delivered to hydrogenases via RrFNR and SynFd (Koo et al., 2016). In this study, we replaced the source of O_2_ from air to 10 vol% ^18^O_2_. This allowed us to directly measure accumulation of H_2_^18^O in the buffer using mass spectrometry. Since Fd^*red*^ was reported to be capable of only uni- and divalent reduction of O_2_ (Allen, 1975), we modified the biochemical reaction as described in Figure 1. Instead of adding the hydrogenase enzyme for the NADPH-driven H_2_ production (blue arrows from Fd^*red*^), SOD and catalase were added in the absence of the hydrogenase (red arrows from Fd^*red*^) to increase the likelihood that any reactive oxygen species (ROS) generated by Fd^*red*^ would be converted into H_2_^18^O for isotopic detection.

**FIGURE 2:**
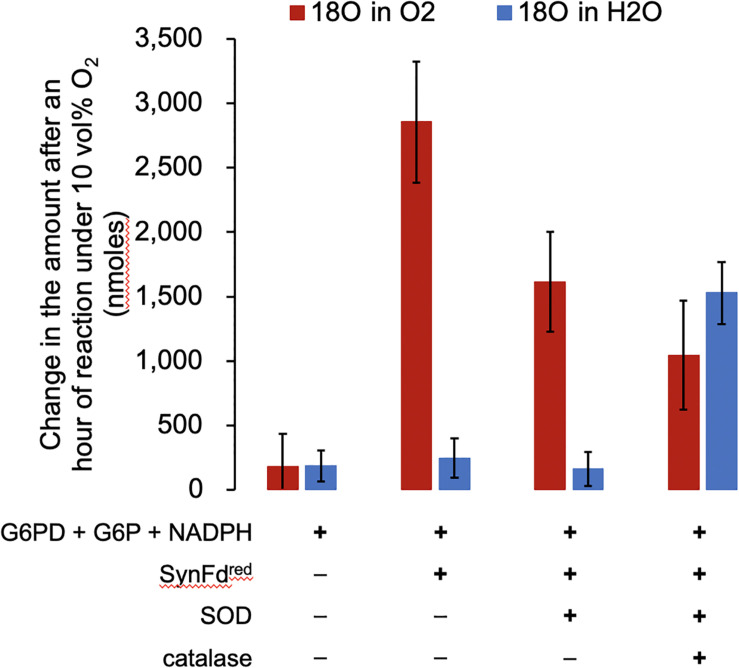
The amount of ^18^O_2_ consumed and H_2_^18^O generated by SynFd^*red*^ over an hour with 10 vol% O_2_ in the headspace. The error bars represent standard deviation (*n* = 2).

Four reaction mixtures were prepared, incubated with 10 vol% ^18^O_2_ for an hour, and analyzed to determine (1) consumption of the headspace ^18^O_2_ and (2) formation of H_2_^18^O. The concentrations of reagents were as follows: 10 mM G6P, 4 U G6PD, 5.0 mM NADPH, 5.0 μM RrFNR, 5.0 μM SynFd, 4 U SOD, and 4 U catalase in 50 mM Tris buffer, pH 7.0. Incremental addition of reagents in reaction sequence (Figure 1) starting at SynFd clearly confirmed our previous finding (Koo et al., 2016): SynFd^*red*^ is responsible for O_2_ consumption (Figure 2). The results also suggested that SynFd^*red*^ is not capable of reducing O_2_ fully into H_2_O. This was evident from the observation that a significant amount of H_2_^18^O was formed only in the presence of both SOD and catalase. The fact that *Synechocystis* sp. cells have native SOD and catalase is consistent with our interpretation in that these enzymes can rapidly remove harmful O_2_ radicals generated by SynFd^*red*^.

Assuming that SOD do not impact the O_2_ reduction activity of SynFd^*red*^, we expected to see the decrease in headspace O_2_ reduce by half (Figure 1, following the red arrows only from Fd^*red*^) in the mixture containing only up to SOD. This is indeed what we observed: The loss of headspace O_2_ after an hour decreased from roughly 2,900 to 1,600 nmoles upon addition of only SOD, approximately 50% reduction considering experimental error. We also expected to see a further reduction in the amount by which headspace O_2_ decreases upon addition of catalase to the mixture containing SOD (Figure 1) since O_2_ is regenerated in the presence of both. Indeed, the loss of headspace O_2_ after an hour further decreased to roughly 1,000 nmoles. The water molecules containing ^18^O were only formed when both SOD and catalase were added to the reaction mixture. Furthermore, the amount of H_2_^18^O produced, 1,500 nmoles, is similar to the stoichiometrically expected value of 1,300 nmoles: Each peroxide molecule generated by SOD will be converted into a water molecule by catalase. Based on the headspace O_2_ loss, the average turnover number (TON) of O_2_ reduction by SynFd was 11 per minute per reduced ferredoxin. It is important to note that this TON is not physiologically relevant. Fd mostly delivers electrons for NADPH regeneration inside cells while, in our experiments, the SynFd molecules were reduced using the electrons extracted from NADPH to consume O_2_.

### Kinetic Study on O_2_ Reduction by Fd^*red*^

We next examined how the rate of electron loss to O_2_ varies with respect to O_2_ partial pressure in the headspace. As discussed in the previous section, SOD and catalase affect the apparent O_2_ consumption by regenerating about half of the putative O_2_ radicals back to O_2_. We thus conducted the following experiments in the absence of SOD and catalase. The concentrations of reagents inside the sealed glass vials were as usual except for RrFNR, which was increased by 10-fold to 50 μM RrFNR. The higher [RrFNR] was chosen in order to enhance electron flux to Fd (*K*_*M*_ of 18 or 39 μM for SynFd and CpFd, respectively) and thereby increase changes in the O_2_ peaks for more accurate analysis.

**FIGURE 3:**
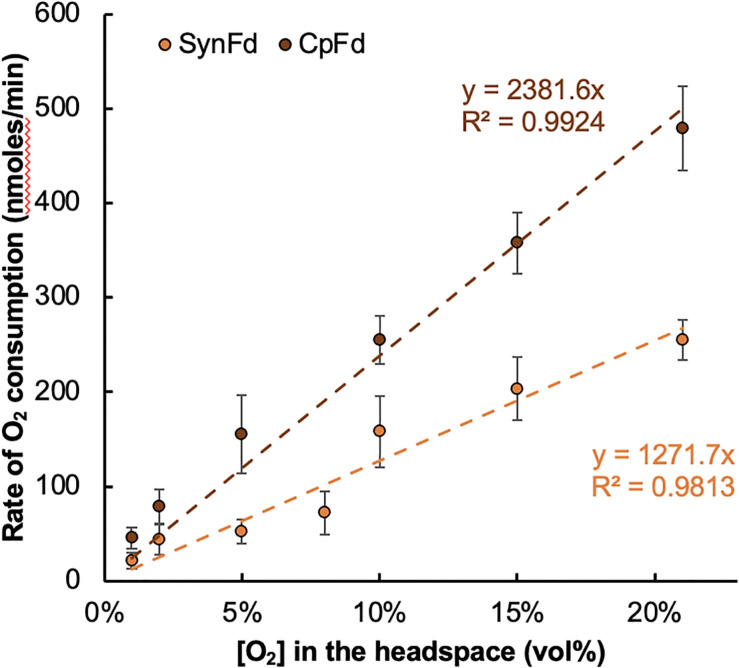
Changes in the rate of O_2_ reduction by SynFd^*red*^ and CpFd^*red*^ versus the headspace [O_2_] inside the reactor vials. Each rate was measured by calculating the linear slope from the time-course of [O_2_] changes in the headspace over the course of 30 min (time interval of 10 min).

The results summarized in Figure 3 and Supplementary Figure 1 indicated that the O_2_ reduction rate, or equivalently the electron loss rate is directly proportional to the headspace [O_2_]. A linear relationship between the two was observed for both types of Fd from 1 to 21 vol% O_2_ in the headspace of the reactor vial. Within this range, the observed O_2_ consumption rate varied from 21 to 255 or 46 to 479 nmoles/min for SynFd and CpFd, respectively. The corresponding specific rate of electron loss to O_2_ ranged between 5 and 61 per minute for SynFd (assuming only univalent reduction of O_2_) and approximately 1.9-fold higher for CpFd. As expected, increasing the [RrFNR] from 5 to 50 μM increased the specific rate of electron loss, but only by about three-fold.

Based on these observations, we analyzed the kinetic parameters for the reductive O_2_ consumption. The following rate law (Eq. 1a) was proposed where the consumption is modeled as a non-enzymatic biochemical reaction between Fd^*red*^ and O_2_:

(1a) $$\frac{d\,\left[O_{2}\right]}{d\,t}=-k_{1}\,{\left[F\,d^{r\,e\,d}\right]}^{a}\,{\left[O_{2}\right]}^{b}$$

(1b) $$\frac{d\,\left[O_{2}\right]}{d\,t}=-k_{1^{\prime }}\,{\left[O_{2}\right]}^{b}$$

where [O_2_] refers to the concentration of O_2_ in the aqueous buffer solution. For the cases where [Fd^*red*^] is fixed, the rate law further simplifies into the equation only dependent on [O_2_] (Eq. 1b). This will be true if the electron flux from FNR to Fd increases as [O_2_] increases to maintain [Fd^*red*^] approximately constant. We observe a linear fit for the plot of *dO*_2_/*dt* versus [O_2_] (Figure 3), which suggests that the O_2_ reduction by Fd^*red*^ follows a pseudo first order rate law under the experimental conditions. The rate constant of this kinetic model (eqn. 2, *b* = 1), namely $k_{1^{\prime }},$ is calculated to be 0.0013 and 0.0025 per minute for SynFd and CpFd, respectively.

A multitude of factors may be responsible for the differences in the O_2_ reduction activity of the two types of Fd. To begin with, SynFd harbors one [2Fe–2S] cluster while there are two [4Fe–4S] clusters in CpFd (Bertini et al., 1995; Van Den Heuvel et al., 2003). The former is known to be capable of transitioning between [2Fe–2S]^2+^ and [2Fe–2S]^+^ while the latter can undergo changes among three states—[4Fe–4S]^3+^, [4Fe–4S]^2+^, and [4Fe–4S]^+^ (Yao et al., 2012). The midpoint redox potentials (RE) are similar: −412 and −387 mV for SynFd and CpFd, respectively (Grzyb et al., 2018). The dissociation constants with RrFNR and CpI are also not significantly different. In this regard, the difference in the range and nature of oxidation states appear to be more important with respect to the Fd’s O_2_ reduction activity. The results in the subsequent section suggests that the local environment surrounding the Fe–S clusters also matter.

### Aerobic H_2_ Production From Various Pairs of FNR and Fd

We have previously reported that the rate of O_2_ consumption is different when SynFd versus CpFd is used for the NADPH-driven H_2_ production pathway (Koo et al., 2016). The rate was approximately twice as fast with CpFd, meaning that more electrons are lost to O_2_ reduction instead of being used for H_2_ production by the hydrogenase. The choice of CpFd over SynFd would therefore result in a lower NADPH-driven H_2_ production efficiency. In this manner, the choice of Fd can impact the efficiency and overall yield of aerobic, biological H_2_ production when Fd molecules deliver electrons to the hydrogenase enzyme.

We examined various combinations of Fd and FNR to study how the electron loss to O_2_ differs during H_2_ production (Figure 1, co-existence of both the blue and red arrow pathways) under 5.0 vol% O_2_. Different FNRs were evaluated as well since the electron flux rate through the FNR could influence the steady state [Fd^*red*^] (Bingham et al., 2012; Lu et al., 2015), which in turn would affect the rate of electron loss to O_2_. The concentrations of the reagents were as follows: 10 mM G6P, 1 U G6PD, 5 mM NADPH, 5 μM FNR, 5 μM Fd, and 10 nM WT CpI in 50 mM Tris–HCl buffer pH 7.0. The reaction lasted for an hour, and we analyzed the data with respect to (1) anaerobic FNR TON during H_2_ production, (2) relative aerobic H_2_ yield, and (3) the overall change in the headspace [O_2_].

The results (Table 1) indicated that electrons in Fd^*red*^ are lost to O_2_ at a faster rate with a higher (anaerobic) FNR TON within the NADPH-driven H_2_ production (*r* = 0.82). This was expected since a higher FNR TON means a greater electron flux to Fd. In contrast, there was insignificant correlation between the anaerobic FNR TON and the relative aerobic H_2_ yield of the reaction (*r* = −0.25). This confirmed our previous finding from the CpI mutagenesis study where we reported insignificant relationship between the two variables (Koo and Swartz, 2018). This observation suggests that it may be possible to improve both the electron flux and aerobic H_2_ yield by engineering FNR and/or Fd. Table 1 also revealed that AnFd is the most efficient in delivering electrons to the hydrogenase in the presence of O_2_. In contrast, CpFd lost the most electrons to O_2_ among the four types of Fd studied in this experiment. The difference in efficiency between the two ferredoxins was striking: The overall amount of O_2_ consumed by CpFd^*red*^ over an hour was as much as four-fold of the amount by AnFd^*red*^.

**TABLE 1:** The anaerobic FNR TON, the relative aerobic H_2_ yield, and the amount of reductive O_2_ consumption over 1 hour for various combinations of FNR-Fd under 5.0% O_2_.

FNR	SynFNR	RrFNR	SynFNR	RrFNR	SynFNR	RrFNR	SynFNR	RrFNR
**Fd**	**SynFd**		**CpFd**		**ZmFd**		**AnFd**	
FNR TON (min^–1^)	5.2	4.4	5.0	10.5	2.7	4.9	3.8	6.0
H_2_ production μmoles)	0.11	0.07	0.09	0.16	0.06	0.09	0.07	0.16
H_2_ yield (%)^*a*^	8.5	6.7	6.8	6.1	8.1	7.0	7.3	10.4
△O_2_ (μmoles)	0.9	1.8	1.8	3.3	1.0	1.5	0.7	1.0
e^–^ flux to O_2_ reduction^*b*^ (%)	28	54	51	50	46	48	34	24

*^*a*^The relative aerobic H_2_ yield refers to the percent of expected production based on the anaerobic production rate. For example, for the combination of SynFNR and SynFd, the anaerobic FNR TON is 5.2 per minutes and the amount of H_2_ produced anaerobically over an hour is about 1.3 μmoles; the aerobic H_2_ yield of 8.5% means that 0.1 μmoles are produced instead in the presence of 5.0 vol% O_2_ in the headspace. For further explanations, please refer to our previous work (Koo et al., 2016). ^*b*^This is calculated based on the assumption of univalent O_2_ reduction by Fd.*

AnFd harbors one [2Fe–2S] cluster with the midpoint RE potential of −405 mV (Jacobson et al., 1993). This is not much different from ZmFd, for example, which also features one [2Fe–2S] cluster and the midpoint RE potential of −390 mV (Shinohara et al., 2017). The difference in terms of electron leakage to O_2_ reduction during H_2_ production, however, is nearly two-fold. The slightly higher midpoint RE cannot explain this difference since SynFd loses more electrons to O_2_ in spite of the lower midpoint RE potential (−412 mV) than AnFd (Table 1). These results indicate that other factors affect the partitioning of the electron flux between H_2_ production and O_2_ reduction. Given the substantial difference for the same type of Fd when a different FNR is used, we suspect that the binding dynamics is one of the factors influencing the partitioning. The reported binding affinities between native Fds and FNRs used in this study range between 15 to 50 μM (Lu et al., 2015; Shiigi, 2015). No obvious relationships between these affinities and the FNR TON or electron flux to O_2_ reduction were observed, likely owing to the complex nature of multi-component interactions in our assay (Figure 1). A fully exhaustive study on the binding interactions between each pair within the assay may help to elucidate the electron partitioning phenomena.

## Conclusion

We studied how the choice of ferredoxin affects the aerobic, biological H_2_ production catalyzed by the [FeFe] hydrogenase molecules. The results revealed that the electron flux from the reduced ferredoxin can partition between O_2_ and the hydrogenase and that the ratio of partitioning can vary among the different types of ferredoxin. We compared the results obtained by combining two different types of FNR with four different types of Fd. The differences in the amount of O_2_ reduced and H_2_ produced over an hour were as large as 4.7- and 2.3-fold of the lowest set. Our findings suggest that it is possible to reduce the electron loss to O_2_ by varying Fd and/or FNR within the biological H_2_ production pathway. The results also hint the possibility of simultaneously improving the TON of H_2_ production (which is limited by FNR TON in the NADPH-driven reaction). Since the two redox proteins are commonly found across many forms of life, large combinatorial libraries may be screened to find even better pair to enhance biological H_2_ production beyond the current state of the art.

## Data Availability Statement

The original contributions presented in the study are included in the article/Supplementary Material, further inquiries can be directed to the corresponding author.

## Author Contributions

JK planned and conducted research. He also wrote the manuscript, analyzed data. YC conducted part of the experiments and also contributed to analysis of some of the data. Both authors contributed to the article and approved the submitted version.

## Conflict of Interest

The authors declare that the research was conducted in the absence of any commercial or financial relationships that could be construed as a potential conflict of interest. The handling editor declared a past co-authorship with one of the authors, JK.
