# Supplementary material for: Investigation of the Ferredoxin’s Influence on the Anaerobic and Aerobic, Enzymatic H2 Production
Source: Front Bioeng Biotechnol. 2021 Feb 26;9:641305. doi: 10.3389/fbioe.2021.641305 (PMC7952640; doi:10.3389/fbioe.2021.641305)
Supplement: Supplementary file 1 [file Data_Sheet_1.docx]

Supporting Information for

**Investigation of the ferredoxin's influence on the anaerobic and aerobic, enzymatic H_2_ production**

Jamin Koo^1,*^, Yeeun Cha^1^

^1^Department of Chemical Engineering, Hongik University, Seoul, 04066, Republic of Korea

**Table of Contents**

**Figure S1**. The time course of changes in the headspace O_2_ due to reduction by the reduced SynFd within the NADPH-driven assay.

**Figure S1**. The time course of changes in the headspace O_2_ due to reduction by the reduced SynFd within the NADPH-driven assay. The error bars represent the standard deviation of duplicate experiments performed on separate days. The O_2_ reduction rate shown in Figure 1 of the main text were calculated by taking the linear slope between the first two time points in these graphs.
